# Identification of Selection Footprints on the X Chromosome in Pig

**DOI:** 10.1371/journal.pone.0094911

**Published:** 2014-04-16

**Authors:** Yunlong Ma, Haihan Zhang, Qin Zhang, Xiangdong Ding

**Affiliations:** Key Laboratory of Animal Genetics, Breeding and Reproduction, Ministry of Agriculture, National Engineering Laboratory for Animal Breeding, College of Animal Science and Technology, China Agricultural University, Beijing, P.R. China; Wageningen UR Livestock Research, Netherlands

## Abstract

Identifying footprints of selection can provide a straightforward insight into the mechanism of artificial selection and further dig out the causal genes related to important traits. In this study, three between-population and two within-population approaches, the Cross Population Extend Haplotype Homozygosity Test (XPEHH), the Cross Population Composite Likelihood Ratio (XPCLR), the F-statistics (Fst), the Integrated Haplotype Score (iHS) and the Tajima's D, were implemented to detect the selection footprints on the X chromosome in three pig breeds using Illumina Porcine60K SNP chip. In the detection of selection footprints using between-population methods, 11, 11 and 7 potential selection regions with length of 15.62 Mb, 12.32 Mb and 9.38 Mb were identified in Landrace, Chinese Songliao and Yorkshire by XPEHH, respectively, and 16, 13 and 17 potential selection regions with length of 15.20 Mb, 13.00 Mb and 19.21 Mb by XPCLR, 4, 2 and 4 potential selection regions with length of 3.20 Mb, 1.60 Mb and 3.20 Mb by Fst. For within-population methods, 7, 10 and 9 potential selection regions with length of 8.12 Mb, 8.40 Mb and 9.99 Mb were identified in Landrace, Chinese Songliao and Yorkshire by iHS, and 4, 3 and 2 potential selection regions with length of 3.20 Mb, 2.40 Mb and 1.60 Mb by Tajima's D. Moreover, the selection regions from different methods were partly overlapped, especially the regions around 22 ∼25 Mb were detected under selection in Landrace and Yorkshire while no selection in Chinese Songliao by all three between-population methods. Only quite few overlap of selection regions identified by between-population and within-population methods were found. Bioinformatics analysis showed that the genes relevant with meat quality, reproduction and immune were found in potential selection regions. In addition, three out of five significant SNPs associated with hematological traits reported in our genome-wide association study were harbored in potential selection regions.

## Introduction

Artificial selection plays an important role in the process of adaptive evolution of domestic animals [1]. So far, a series of noticeable differences caused by artificial selection have been identified, especially the economic traits which brought huge economic profit in the development of human society [1], [2]. With the development of high throughput genotyping technology, hunting genomic evidence of selection on genes or genomic regions via high-density SNP chips or sequencing data shows useful to provide straightforward insights into the meaning of selection and explore causal genes relevant to traits of interest [3], [4].

Theoretically, a novel causal variant that has been under the pressure of selection usually shows long-range linkage disequilibrium (LD) and a high population frequency over a long period of time. Hence, selection footprints could be detected through the decay of linkage disequilibrium and the variation of allele frequency. So far, a series of related methods have been proposed and can be grouped into categories of site-frequency spectrum and linkage disequilibrium according to the theory of them [5]. The F-statistics (Fst) [6], the Tajima's D test [7], and the Cross Population Composite Likelihood Ratio (XPCLR) [8], the Cross Population Extend Haplotype Homozygosity Test (XPEHH) [4] and the Integrated Haplotype Score (iHS) [9], as the representative method respectively corresponding to each category, are widely used in identifying selection footprints. Among them, Fst, XPCLR and XPEHH are mainly used to detect selection footprints between populations (between-population methods), both the Tajima's D and iHS are primarily using the information from single population to reveal the selection footprints (within-population methods). Fst was initially used to assess the population differentiation according to the DNA polymorphism of populations [6], which was attributed to the geographically variable selection. Currently, some branches of Fst methods have been developed, e.g. the two-step method of Gianola's Fst [10], Fst-based Bayesian hierarchical model [11]. Different from Fst, the XPCLR uses the differentiation of multi-locus allele frequency between two populations to detect selection footprints, it is effective in identifying the fast changes in allele frequency at the locus with random drift [8]. The major consideration of Fst and XPCLR is the variation of allele frequency while XPEHH assumes that the occurrence of selection can be traced through measuring LD or observing overrepresented haplotype in population, making it capable to detect entirely or approximately fixed site [4]. The iHS is also based on theory of linkage disequilibrium, it is sensitive for finding the regions with a rapidly increased frequency of the derived allele at selected sites [9]. Tajima's D is based on allele frequency and it is sensitive to purifying selection and balancing selection [7].

At present, many studies of selection footprints in human and animals were reported [3], [4], [12]–[15]. However, most of these studies circumscribe the investigations on the autosomes and rarely on the X chromosome. Comparing with autosome, the X chromosome has its own particularity and plays an important role in evolution of human and animals, McVicker et al. (2009) investigated the genomic signature of natural selection and found that genome diversity reduction caused by selection on the X chromosome (12–40%) is higher than on the antosomes (19–26%) [13]. The X chromosome has suffered higher selection pressure than autosomes due to the sex-specific dosage compensation (SSDC),resulting in genes on the X chromosome under more direct and effective selection [16], [17]. As an important model animal, pig has experienced a long history of artificial selection in the process of domestication and breeding [18]. The X chromosome of pig carries many interesting genes like androgen receptor gene (AR) and thyroid-binding globulin gene (TBG). Therefore, it is necessary to investigate the occurrence of selection footprints on the X chromosome in pig.

In this study, three between-population methods (XPEHH, Fst and XPCLR) and two within-population methods (iHS and Tajima's D) were implemented to scan the whole X chromosome for hunting selection footprints in three pig breeds through Illumina PorcineSNP60K BeadChip (Illumina, San Diego, CA). Afterwards, a stream of analysis, including gene searching and functional enrichment analysis, were performed to explain the biological significant of selection footprints.

## Materials and Methods

### Experiment Animals

A total of 515 pigs were selected out from three breeds as the experimental population in this study. There are 67 individuals (32 boars and 35 sows) in Landrace, 375 individuals (207 boars and 168 sows) in Yorkshire and 73 individuals (39 boars and 34 sows) in Chinese Songliao (Songliao for short). Songliao was bred in 1988 using boars of cross-bred of Duroc and Landrace, and sows of Minzhu, one famous Chinese native breed.

In order to identify population structure and avoid the relatedness of animals, the principal component analysis (PCA) followed Paschou et al. (2007) was performed using the genotype data [19]. A total of 113 sows, including 35 from Landrace, 34 from Songliao and 44 from Yorkshire, were finally chosen to detect selection footprints on the X chromosome. As shown in Fig. S1, the contribution of first principal component captures 36.14% of the variance in this data, and the second 22.03%.

### Genotyping Data

Genomic DNA samples were extracted from ear tissue of all 515 pigs. The whole procedure for collecting ear tissue samples was carried out in strict accordance with the protocol approved by the Animal Welfare Committee of China Agricultural University (Permit Number: DK996). All DNA samples were genotyped using the Infinium II Multisample assay (Illumina Inc.). Illumina Porcine60K SNP arrays were scanned using iScan (Illumina Inc.) and analyzed using BeadStudio (Version 3.2.2, Illumina, Inc.).

We implemented one procedure to ensure the high quality of genotyping on the X chromosome of all sows: (1) the individuals with call rate less than 0.90 were discarded; (2) SNP loci were removed on condition that the SNP call rate <0.90; (3) SNP loci severely deviated from Hardy-Weinberg equilibrium (p-value <10e–6) were removed; (4) SNP loci with minor allele frequency (MAF) less than 0.05 were removed only when within-population methods were performed. After quality control, we imputed the missing genotypes and inferred haplotypes using BEAGLE [20].

### Detection of Selection Footprints

Three between-population methods (XPEHH, Fst and XPCLR) and two within-population methods (iHS and Tajima's D) were implemented to detect the selection footprints. Fst, XPCLR and Tajima's D can directly handle SNP genotype, while XPEHH and iHS mainly use phased data.

#### Calculation of XPEHH scores

The XPEHH derives from the idea of Extended Haplotype Homozygosity (EHH), which is defined as a probability that two randomly chosen extended haplotypes carrying a given core haplotype are homozygosity [3], [4], [21], EHH is calculated as 
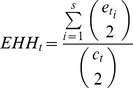
(1)where 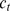 is the number of sample of a particular core haplotype 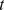, 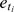 is the number of samples of a particular extended haplotype 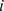 which is based on a particular core haplotype 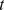 and 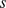 is the number of unique extended haplotypes.

The basic idea of XPEHH is to test if the site is homozygous in one population but polymorphic in another population through the comparison of EHH score of two populations on one core SNP. It is expressed as 
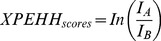
(2)


where 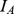 is the integral of the EHH value with respect to genetic distance in population A, 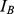 is in population B. Population B is viewed as reference population and population A as observed population. Negative XPEHH score suggests selection happened in reference population, otherwise in observed population. XPEHH is highly powerful in detecting those with approximately fixed or fixed selected loci [3].

#### Population differentiation index

As a single locus analysis method, Fst generally quantifies the relationship between pairs of the allele within subpopulation and the meta-population for measuring the degree of differentiation. In this study, a two-steps process proposed by Gianola et al. [10] was employed to identify selection footprints based on population differentiation. In the first step, with a non-informative prior distribution of allelic frequency, a method to model the Bayesian drawing samples from the posterior distribution of 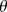 parameters was introduced. According to Bayes theorem, the joint posterior density of all allelic frequencies is 
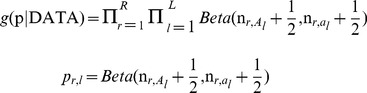
(3)where *R* represents the total number of subpopulations; 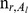 represents the frequency of allele A at 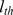 site in subpopulation; 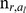
represents the frequency of allele a at 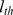 site in subpopulation. The second step, considering the posterior distribution samples as “data”, goes to model the finite mixture to figure out the clusters of 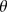 statitics. Then, a draw from the posterior distribution of 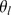 is expressed as
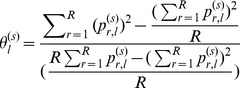
(4)where the mean posterior distribution of 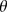 (Fst) value between populations ranges from 0 (identical population) to 1 (complete differentiation).

#### Calculation of XPCLR values

To avoid the influence of SNP ascertainment bias, XPCLR was built upon the multiple-locus composite likelihood ratio method (CLR) [8]. It not only makes use of the differences in allele frequencies between populations, but also models the joint allele frequency spectrum under selection. The likelihood function is given by 
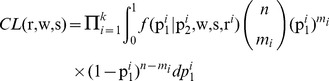
(5)where *r* is vector of recombination rate: 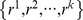, *n* is the sample size, 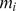 stands for the count of neutral allele at locus *i*, *s* is the selection coefficient, *k* is the size of sliding window, w is a weight factor on linkage disequilibrium and *p* represents the allele frequency.

#### Calculation of iHS scores

The iHS statistic was defined as the log of the ratio of the integrated EHH score for haplotypes centering the ancestral allele to the integrated EHH score for haplotypes centering the derived allele as described by Voight et al. (2006) [9]. The standardized iHS is defined as 
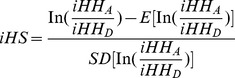
(6)Where 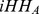 and 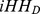 represent the integrated EHH score for ancestral and derived core allele. The final statistic approximately follows a standard normal distribution [9].

#### Calculation of Tajima's D

The **Tajima's D** test considers the difference between the mean pairwise difference 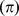 and the number 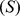 of segregating sites in nucleotide polymorphism data [7]. It is expressed as:
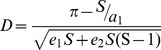
(7)where 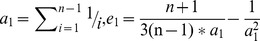 and n is the number of sequences. The statistic equals zero for neutral variation, and is negative when an excess of rare polymorphism caused by a recent selective sweep and is positive with the excess of high-frequency variants suggests balancing selection for multiple alleles.

#### Identifying potential selection footprints

Separately for each population/population pair analysis, two different procedures were implemented to determine the significance of statistic values and to identify potential selection footprints. (1) For XPEHH, iHS and XPCLR, which can make use of multiple markers, followed Voight et al. (2006) [9], the thresholds of empirical cutoffs for the X chromosome were based on the autosomal cutoffs. We determined empirical cutoffs for the top 5% of signals genome-wide on all autosomes, the statistic values on the X chromosome that were above these thresholds were considered to be outlier and treated as potential selection footprints. (2) For Fst and Tajima's D, we implemented 5000 permutation tests to establish the empirical distributions of Fst and Tajima's D. As describe by Qanbari et al. [22], in each permutation test, we shuffled the allele frequencies randomly across the fixed SNP positions. The threshold values at significance level of 0.05 from the empirical distribution were used to determine the significance of statistic. In addition, we also carried out 5000 permutation tests on XPCLR to see the plausibility of permutation test on approaches handling multiple markers.

### Bioinformatics Analysis

Based on the findings from detection of selection footprints, further bioinformatics analyses were carried out to reveal the potential biological function of genes harbored in selection regions.

#### Enrichment analysis

The process of enrichment analysis, including cellular component, molecular function, biological process and the KEGG pathway, was performed for the candidate selection regions. Considering only quite few available annotation on pig genome, the abundant database of human genomic information was referred to identify genes on pig genome. The program of BioMart (http://www.biomart.org/)[23] and DAVID 6.7 (http://david.abcc.ncifcrf.gov/) [24]were employed to generate the homology gene set and gene enrichment analysis.

#### Gene annotation

In the analysis, the interest region (so-called selection region) for annotation is empirically defined as the chromosome segment, in which the outlier or selection footprint was extended about 400 kb towards its upstream and downstream directions. According to the selection regions, we identified the particular biological function through the database of NCBI (http://www.ncbi.nlm.nih.gov/gene/). In addition, we validated those regions with the candidate regions found in our previous genome-wide association study (GWAS) research [25].

## Results

### Information of Markers

After quality control and principal component analysis, 35, 34, 44 individuals and 1163, 1136 and 1159 SNPs corresponding to Landrace, Songliao and Yorkshire were finally retained in this analysis. In order to implement three between-population methods XPEHH, Fst and XPCLR, 1129, 1146 and 1132 common SNPs were separately chosen from the pairs of Landrace-Songliao (L-S for short), Landrace-Yorkshire (L-Y) and Yorkshire-Songliao (Y-S). The average distance of adjacent SNPs corresponding to three breed pairs is 111.60 kb, 109.95 kb and 111.31 kb, respectively.

### Empirical Distribution of Test Statistic

**Figure 1:**
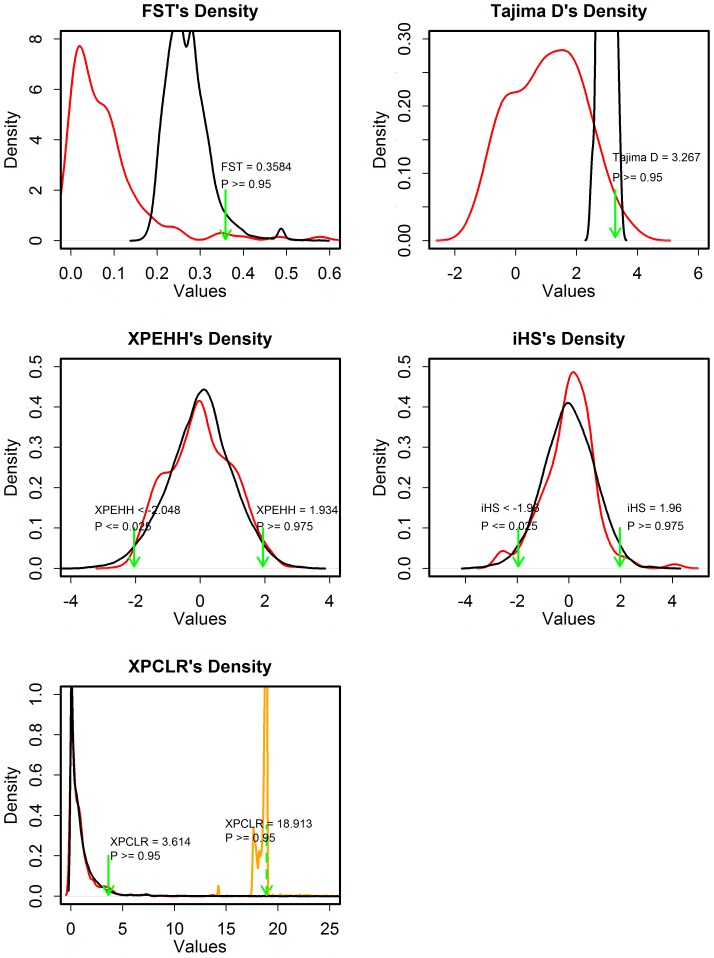
Posterior density of five test statistics. The distributions of five test statistics on the X chromosome (red line), empirical distributions of Fst, Tajima's D (black line) and XPCLR (yellow line) from 5000 permutation tests, and the distributions of XPEHH, iHS and XPCLR on all autosomes (black line), respectively. Tajima's D and iHS are for Landrace, XPEHH, XPCLR and Fst are for breed pair of Landrace-Yorkshire only.

The distributions of test statistics of three between-population methods for each breed pair and of two within-population methods for each breed can be clearly illustrated. Taking Landrace and breed pair of Landrace-Yorkshire (L-Y) for instance, Fig. 1 plots the distributions of these five test statistics on the X chromosome data (red line), empirical distributions of Fst, Tajima's D (black line) and XPCLR (yellow line) from 5000 permutation tests, and the distributions of XPEHH, iHS and XPCLR on all autosomes (black line), respectively. The distributions of XPEHH and iHS on the X chromosome are nearly in accordance with their distributions on autosomes, and these two test statistics from autosomes data more follow standard normal distribution, as pointed out by Sabeti et al. [4]. Correspondingly, the critical value for iHS at significance level of 0.05 are 1.96 and −1.96, and those for XPEHH are very close to standard normal distribution with 1.934 and −2.082. For Fst and Tajima's D, their critical values from empirical distributions are much stricter, making the detection of selection footprints more convinced. Two procedures were used to generate critical values for XPCLR, while the critical value from permutation test is so high that no selection footprints were detected. The distribution of XPCLR on the X chromosome is nearly as same as that on all autosomes, therefore the critical value from autosomal cutoffs is more reasonable and used in our whole study. In addition, the distributions of the five test statistics indicate similar tendency for other breeds and breed pairs (Fig. S2 and Fig. S3).

### Selection footprints and regions detected by between- and within-population methods

Table 1 summaries the selection footprints which were identified in three breed pairs (L-Y, L-S and S-Y) by three between-population methods, respectively. For breed pair of Landrace-Songliao (L-S), 27 negative values out of 64 outliers suggest that selection happened in the reference population of Songliao, and the other 37 outliers indicate that selection happened in Landrace when XPEHH test was used. Similarly, 32 outliers were detected in Landrace-Yorkshire (L-Y) with 5 selection happened in Yorkshire and 27 in Landrace. Hence, 64 selection footprints, including 37 outliers from L-S pair and 27 outliers from L-Y, were revealed in Landrace in total. Likewise, 72 and 34 selection footprints were detected in Songliao and Yorkshire, respectively (Table 1).

**Table 1:** Summary of selection footprints detected by three between-population methods in different pig breed pairs.

Breed pair^1^	Number of SNP	Average SNP density (kb)	XPEHH	XPCLR	Fst
**L-S**	1129	111.60	64(L 37, S 27)^2^	59(S 41, L 18)^2^	1(L 1, S 1)^2^
**L-Y**	1146	109.95	32(L 27, Y 5)	60(Y 31, L 29)	3 (L 3, Y 3)
**Y-S**	1132	111.31	74(Y 29, S 45)	70(S 33 Y 37)	1 (Y 1, S 1)

1 L-S represents breed pair of Landrace and Songliao, Y represents Yorkshire.

2 The number of selection footprints (selection region for Fst) separately identified in two breeds for one breed pair in brackets.

For the implementation of Gianola's Fst, the whole X chromosome was divided into a series of non-overlapping, consecutive, 800-kb windows. The windows, in which SNPs with Fst values higher than the empirical critical value at significance level of 0.05 from permutation test, were treated as potential selection region. One selection region detected by Fst indicates the selection happened in both breeds for one breed pair, e.g. for breed pair of Landrace-Songliao (L-S), one same selection region were detected in Landrace and Songliao (Table 1). Different from XPEHH and Fst, the selection footprints were separately detected by XPCLR in observed population, e.g. for breed pair of Landrace-Songliao (L-S), 41 selection footprints were detected in Landrace when Songliao was regarded as reference population, and 18 in Songliao when Landrace was the reference population (Table 1).

Considering the overlap of selection regions, the selection footprints detected by three between-population methods were merged by single breed (Table 2). Taking Landrace as an example, in total 64 outliers were detected (37 from L-S and 27 from L-Y) by XPEHH, after merging the overlapping selection regions harboring those outliers, 11 selection regions were finally identified. Similarly, 11 and 7 selection regions were detected for Songliao and Yorkshire, respectively. On average, each selection region has the length of 1.42 Mb, 1.12 Mb and 1.34 Mb, and correspondingly contains approximately 16.5, 18.3 and 12.3 SNPs in three breeds, respectively. Likewise, 16, 13 and 17 selection regions were identified by XPCLR in Landrace, Songliao and Yorkshire, respectively, with length of 0.95 Mb, 1.00 Mb and 1.13 Mb and harboring 11.63, 15.46, 13.70 SNPs each on average. For Fst, in total 4, 2 and 4 selection regions were finally found with fixed length of 800 kb and containing 6.25, 6.00 and 7.25 SNPs each on average in Landrace, Songliao and Yorkshire.

**Table 2:** Summary of incorporating selection regions in three pig breeds with three between-population methods and two within-population methods.

		Landrace	Songliao	Yorkshire
**XPEHH**	Number of regions	11	11	7
	Average length (Mb)	1.42	1.12	1.34
	Number of SNP/region	16.50	18.30	12.30
**XPCLR**	Number of regions	16	13	17
	Average length (Mb)	0.95	1.00	1.13
	Number of SNP/region	11.63	15.46	13.70
**Fst** 1	Number of regions	4	2	4
	Number of SNP/region	6.25	6.00	7.25
**iHS**	Number of regions	7	10	9
	Average length (Mb)	1.16	0.84	1.11
	Number of SNP/region	20.10	13.30	21.00
**Tajima**'**s D** 1	Number of regions	4	3	2
	Number of SNP/region	13.25	15.00	11.50

1 Each selection region has fixed length of 800 kb.

Comparing with between-population methods, the detection of selection footprints in one population using within-population methods iHS and Tajima's D were relative simple. As shown in Table 2, after merging the overlapping selection regions, in total 7, 10 and 9 selection regions were identified by iHS in Landrace, Songliao and Yorkshire with length of 1.16 Mb, 0.84 Mb and 1.11 Mb and harboring 20.1, 13.3 and 21.0 SNPs each on average. Likewise, Tajima's D was implemented to detect selection region within one breed in the same way as Fst did in breed pair, 4, 3 and 2 selection regions (balancing selection) were separately identified with fixed length of 800 kb and containing 13.25, 15.0 and 11.5 SNPs each on average, but no positive selection was identified.

### The overlap of selection region

**Figure 2:**
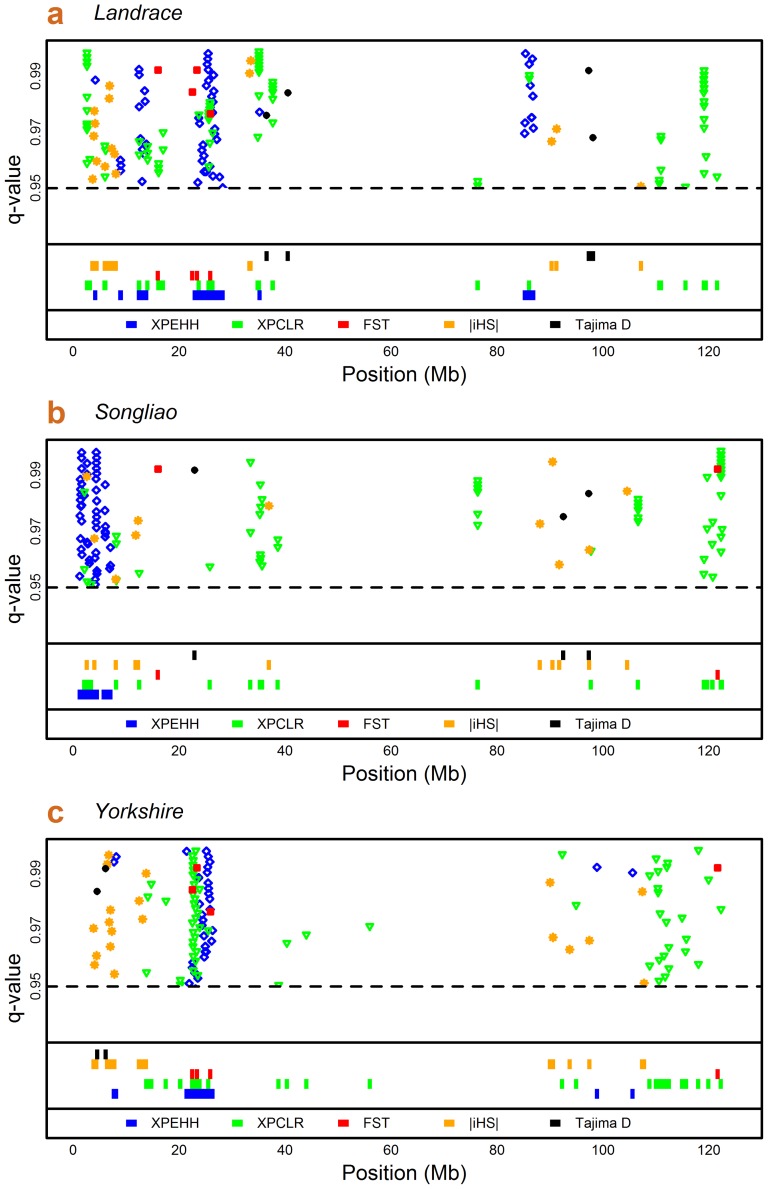
Distribution of selection footprints and selection regions on the X chromosome in three pig breeds. The part above bold line plots the quantile value of selection footprints, and the part below bold line shows the selection regions along the X chromosome. The quantile value is defined as which quartile of the top values of the respective statistic the reported value cuts off.

Fig. 2 presents an intuitive scatter plot, showing the distribution of the quantile values (q-value) of five approaches along physical position on the X chromosome in three breeds, respectively. The majorities of selection footprints in three breeds are concentrated in two ends of the X chromosome and there is a high proportion overlap across different breeds. Table 3 further shows not only the length of selection region identified by five methods respectively, but also the length of overlapping region identified each other. Taking Landrace as an example, the total length of selection regions that were separately detected by XPEHH, XPCLR, Fst, iHS and Tajima's D was about 15.62 Mb, 15.20 Mb, 3.20 Mb, 8.12 Mb and 3.20 Mb. Among them, 3.15 Mb, 1.95 Mb and 1.43 Mb overlapping regions corresponds to the pairs of between-population methods (XPEHH-XPCLR, XPCLR-Fst and XPEHH-Fst). In addition, the overlapping regions between within-population methods and between-population methods are quite few. There is no overlap of selection regions detected by Tajima's D with those detected by XPEHH, XPCLR and Fst. Similarly, only quite small proportion of the selection regions identified by iHS are overlapped with those from XPEHH and XPCLR, and no overlap with Fst.

**Table 3:** Overlap of selection regions (Mb) from five methods in three pig breeds.

		XPEHH	XPCLR	Fst	iHS	Tajima's D
**Landrace**	XPEHH	15.62	3.15	1.43	0.80	0
	XPCLR		15.20	1.95	1.21	0
	Fst			3.20	0	0
	iHS				8.12	0
	Tajima's D					3.20
**Songliao**	XPEHH	12.32	1.88	0	1.29	0
	XPCLR		13.00	0.18	2.77	0
	Fst			1.60	0	0
	iHS				8.40	0.74
	Tajima's D					2.40
**Yorkshire**	XPEHH	9.38	0.56	1.82	0.88	0
	XPCLR		19.21	2.15	0.74	0
	Fst			3.20	0	0
	iHS				9.99	1.16
	XPCLR		15.20	1.95	1.21	0

### The biological function in selection regions

Based on the findings of selection regions, orthologous comparison analysis revealed that a total of 166, 132 and 241 genes were harbored in all selection regions in Landrace, Songliao and Yorkshire, respectively. While the further enrichment analysis using DAVID v2.1 [24] indicated that quite few functional terms were significant after Benjamini or Bonferroni correction (see Table S1). Wang et al. (2012) reported 5 genes on the X chromosome are associated with hematological traits by using the same experiment population as in this study [25]. Among of these 5 genes, three out of them completely fall into and one close to the selection regions were identified in this study. Table 4 presents 5 significant SNPs in their report were involved in 9 selection regions spreading over 3 breeds, respectively. This implies these selection regions might reflect the potential genetic basis of hematological traits in pig. A series of genes not completely harbored in but overlapped with potential selection regions in this study are shown in Table 5. These genes are relevant with reproduction, immune and meat quality based on the gene database in NCBI (http://www.ncbi.nlm.nih.gov/gene/). Among of them, ACE2 with the function of inhibiting the differentiation of adipocytes [26] is overlapped with the potential selection region of 13.06∼13.09 Mb which was identified by XPEHH and iHS, and gene ACSL4 related with meat quality is overlapped with the region of 105.37∼105.45 Mb detected by XPEHH [27]. 2 genes (ATP1B4 and HTR2C), which are overlapped with the potential selection regions in Yorkshire, are relevant with sow reproduction traits, such as infanticide phenomenon and perinatal development of embryo [28], [29]. The other 2 genes (TRPC5 and ZDHHC9) have been reported relevant with disease traits in several studies [30], [31]. Table S2 detailed presents genes located in those potential selection regions detected by at least two methods.

**Table 4:** Selection regions harboring SNPs associated hematological traits reported by Wang et al. (2012).

Position of outlier SNP	Trait	Selection regions (breed)	Max Statistical value (method, q-value)	Candidate gene
**3917606**	Mean corpuscular volume	3418340–4781142 (Y); 3897515–4872159 (S); 3584968–4384968 (S); 3767181–4567181(L); 3234420–4803149(L);	2.32 (iHS, 0.971); 2.57(XPEHH, 0.982); 1.98(iHS,0.943); 2.34(XPEHH.0.988); 2.62(iHS,0.977);	KAL1
**9272275**	red blood cell count	8516875–9391368(L);	2.02(XPEHH,0.960);	LOC100157657
**43443513**	platelet count	40108407–40908406(L);	3.59(Tajima's D,0.984);	LOC100155983
**54837338**	plateletcrit			LOC100516479
**92131194**	platelet count	91878407–92678407 (Y); 92108407–92908406 (S);	7.66(XPCLR,0.998); 3.35(TajimaD,0.975);	LOC100524920

**Table 5:** Some candidate genes located in selection regions.

Position(Mb)	q-value (Statistics, Breed)	Candidate gene	Gene function
**122.170∼122.210**	0.999(XPCLR, S); 0.995(XPCLR, Y);	ZDHHC9	Related with the congenital splay leg [31]
**13.060∼13.090**	0.974(iHS,Y); 0.982(XPEHH,L)	ACE2	Related with the inhibition of the differentiation of adipocytes [39]
**14.053∼14.060**	0.990(iHS,Y); 0.982(XPEHH,L); 0.993(XPCLR,Y)	S100G	Related with the establishment and maintenance of pregnancy in pigs [40]
**7.065∼7.273**	0.996(iHS,Y); 0.986(iHS,L); 0.960(XPEHH,S);	STS	Related with the estrogen actions [41]
**16.290∼16.320**	0.992(FST,S); 0.992(FST,L);	RS1	Related with the X-linked juvenile retinoschisis [42]
**109.824∼109.827**	0.997(XPCLR,Y);	AGTR2	Related with preeclampsia [43]
**1.958∼1.965**	0.998(XPEHH,S); 0.994(XPCLR,S);	OBP	Odorant-binding proteins [44]
**112.770∼112.790**	0.997(XPCLR,Y);	ATP1B4	Plays an essential role in perinatal development [28]
**105.370∼105.450**	0.990(XPEHH,Y);	ACSL4	Related with pork quality [27]
**106.140∼106.280**	0.992(XPCLR,S);	TRPC5	Related with the fight against cardiovascular disease [30]
**108.810∼108.870**	0.995(XPCLR,Y);	HTR2C	Related with infanticide phenomenon [29]

## Discussion

In the past few years, hunting genomic evidence of selection has been widely viewed as an effective approach for exploring the potential genetic mechanism of phenotype polymorphisms and providing more properly interpretation of evolution with the application of high throughput technology [15], [32]. And a series of approaches have been proposed to detect the selection footprint, all approaches have their own strengths and weaknesses. In this study, we employed five representative methods, XPEHH, XPCLR,Fst, Tajima's D and iHS, to explore selection footprints on the X chromosome. Among them, Fst is effective for detecting selection footprints in single locus based on population differentiation [10]. XPEHH was proposed to detect selection footprints with fixed or approximately fixed selection locus [4], XPCLR is more robust in some scenarios as the change in allele frequency occurs too quickly [8]. iHS is effective in detecting ongoing selection footprints, but not in detecting recently compeleted selection footprints [5]. Tajima's D is an traditional and famous within-population method which is sensentive to purifying selection and balancing selection [5]. Furthermore, XPEHH, XPCLR and iHS actually separately find one significant core SNP and grid window by utilizing multiple-locus information, they identified more selection regions than Fst and Tajima's D.

Most of the methods implemented in detecting selection footprints do not follow strict distributions, e.g. XPEHH and iHS just approximately follow normal distribution. Therefore the risk of false positive of the traditional significance test remains high due to the uncertainty of null distribution of test statistic. In addition, the genome-wide scan of selection footprints also brings the dilemma from multiple testing. Permutation test is proved robust and powerful in gene mapping and detection of selection footprints by establishing the empirical distributions of test statistics [22], [33], our results indicate permutation test is plausible for methods only dealing with allele frequency, e.g. Fst and Tajima's D, both methods only utilize the variation of allele frequency. While no selection footprints were detected when implementing permutation test in XPCLR. Once the allele frequencies of fixed SNPs were shuffled through permutation test, the linkage disequilibrium among adjacent SNPs were correspondingly changed, bringing bias as XPCLR mainly make use of information of multiple SNPs. Similarly, permutation test is implausible for haplotype-based method XPEHH and iHS as well, because haplotypes severely depend on linkage disequilibrium of SNPs. In addition, computing time is also demanding for implementation of permutation test in XPEHH and iHS. For multiple SNP methods, Voight et al. (2006) suggested empirical cutoffs using the top 1% or 5% of genome-wide on all autosomes to determine the significance of test statistic [9]. Our results show this strategy is more reasonable and saves computing time.

In addition, the selection footprints identified by mutiple methods, to some extent, are more convinced. Our results indicate there is a high proportion of overlapping selection regions identified by three between-population methods. Particularly, the region around 22 ∼25 Mb was detected under selection in Landrace and Yorkshire by all three between-population methods, while only small part of this region was detect under selection in Songliao by Tajima's D (Fig 2). Unfortunately, the information of genes harbored in these regions is not available so far (Table S2). Comparing with Chinese Songliao, Landrace and Yorkshire share more common genetic background and they have already experienced a relative long period of adaptive evolution, resulting in some genes in these regions nearly fixed, while Songliao was bred through hybridization of Duroc, Landrace and Chinese Minzhu in past three decades. Therefore, this region might imply some genes relevant to the domestication of European and Chinese pigs, it is worth being deep investigated.

Our results indicate LD measured with r^2^ on the X chromosome (0.395 in Landrace, 0.366 in Songliao and 0.381 in Yorkshire) is slightly higher than those on autosomes (0.354 in Landrace, 0.363 in Songliao and 0.344 in Yorkshire), while the SNP density on the X chromosome is about 110 kb but 60 kb on autosomes, it implies that the LD on the X chromosome might be much higher given the same SNP density as autosomes. The genome diversity will be decreased with high LD as reported by McVicker et al. (2009), correspondingly, the genes on the X chromosome will experience higher pressure of selection [13]. At the same time, we also found the potential selection regions gathered around two ends (0–40 Mb and 80–120 mMb) on the X chromosome, especially the end on short arm (0–40 Mb) of the X chromosome suffered more selections, this region was also overlapped with pseudoautosomal region (PAR) of pig, as Skinner (2013) reported PAR in pig was mapped at the beginning of the short arm even the exact position of PAR is not clear so far [34]. The genes in PAR are probably inclined to be suffered higher selection pressure than autosomes attribute to the sex-specific dosage compensation (SSDC) [16], [17]. Meanwhile, there are also some silent regions resulting from X-inactivation on the X chromosome due to the sex-specific dosage compensation (SSDC), resulting in no signal of selection in this region. This could be one explanation to the phenomenon of few selection footprints identified in the central region (∼40–80 Mb) on the X chromosome in our study even the SNP density in this region are nearly equal to that in two ends.

The enrichment analysis to the selection region identified in this study has not find significant terms after correction, while some terms in one test with P-value less than 0.05 indicated their biological information related with hematological traits. For instance, two GO Biological Progresses, including GO:0002035∼brain renin-angiotensin system and GO:0002002∼regulation of angiotensin levels in blood, are corresponding to Yorkshire and Landrace (Table S1). These GO terms imply that some hematological traits might have been suffered selection in the process of evolution and domestication. Coincidently, our findings also indicate that the significant SNPs associated with hematological traits in our previous study [25] are harbored in selection regions, it in some extent suggests that the concerned hematological traits, including RBC (red blood cell count), MCV (mean corpuscular volume), PLT (platelet count) and PCT (plateletcrit), experienced artificial or natural selection. Usually, hematological traits are referred as important index for immune traits. This might indicate the X chromosome plays an important role in immune system of pig as it does in Human [35].

So far, several researches have been carried out to identify selection footprints in pig [2], [15], [32], [36]. Ai et al. (2013) sampled 18 populations with sample size per breed ranged from 5 to 32 [36]. Wilkinson et al. (2013) collected 14 pig breeds with 24–34 individuals per breed [2]. Although these two studies detect selection footprints on the X chromosome besides autosomes using Porcine SNP60 BeadChips, the blending of boars and sows are not reasonable for the analysis of the X chromosome. Rubin et al. (2012) pointed out that the X chromosome should be solely analyzed for the identification of selection footprints [32] and only sows could be used as sex chromosomes and autosomes, even between genders, are subjected to different selective pressures and have different effective population sizes [37]. Obviously, the small sample size per breed from Ai et al. (2013) [36] and Wilkinson et al. (2013) [2] make it unfeasible to use sows only. Amaral *et al*. (2011) carried out whole genome-wide detection of footprints through sequencing of pooled DNA [15], it is more difficult to analyzed the X chromosome separately. Additionly, the pooling size and coverage of sequencing need to take into consideration as point out by Cutler et al. [38]. Herefore, it is worthwhile to use sufficient sows to detect selection footprints on the X chomsome in this study.

## Supporting Information

Figure S1 **Scatter plots of the population structure of 113 individuals via principal component analysis.**
(TIFF)

Figure S2 **Posterior density of five test statistics.** Tajima's D and iHS are for Songliao, XPEHH, XPCLR and Fst are for breed pair of Landrace-Songliao only.
(TIFF)

Figure S3 **Posterior density of five test statistics.** Tajima's D and iHS are for Yorkshire, XPEHH, XPCLR and Fst are for breed pair of Songliao-Yorkshire only.
(TIFF)

Table S1 **The complete list of three breeds' enrichment analysis.**
(XLSX)

Table S2 **Selection regions identified by more than two methods.**
(DOCX)
