# Supplementary figures and images for: Identification of Selection Footprints on the X Chromosome in Pig
Source: PLoS One. 2014 Apr 16;9(4):e94911. doi: 10.1371/journal.pone.0094911 (PMC3989256; doi:10.1371/journal.pone.0094911)

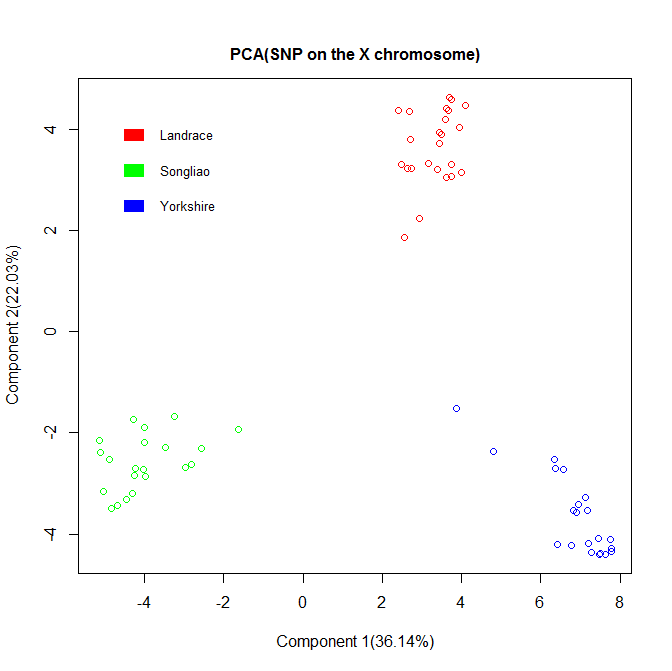

Supplement: Figure S1 — Scatter plots of the population structure of 113 individuals via principal component analysis. (TIFF) [file pone.0094911.s001.tiff]

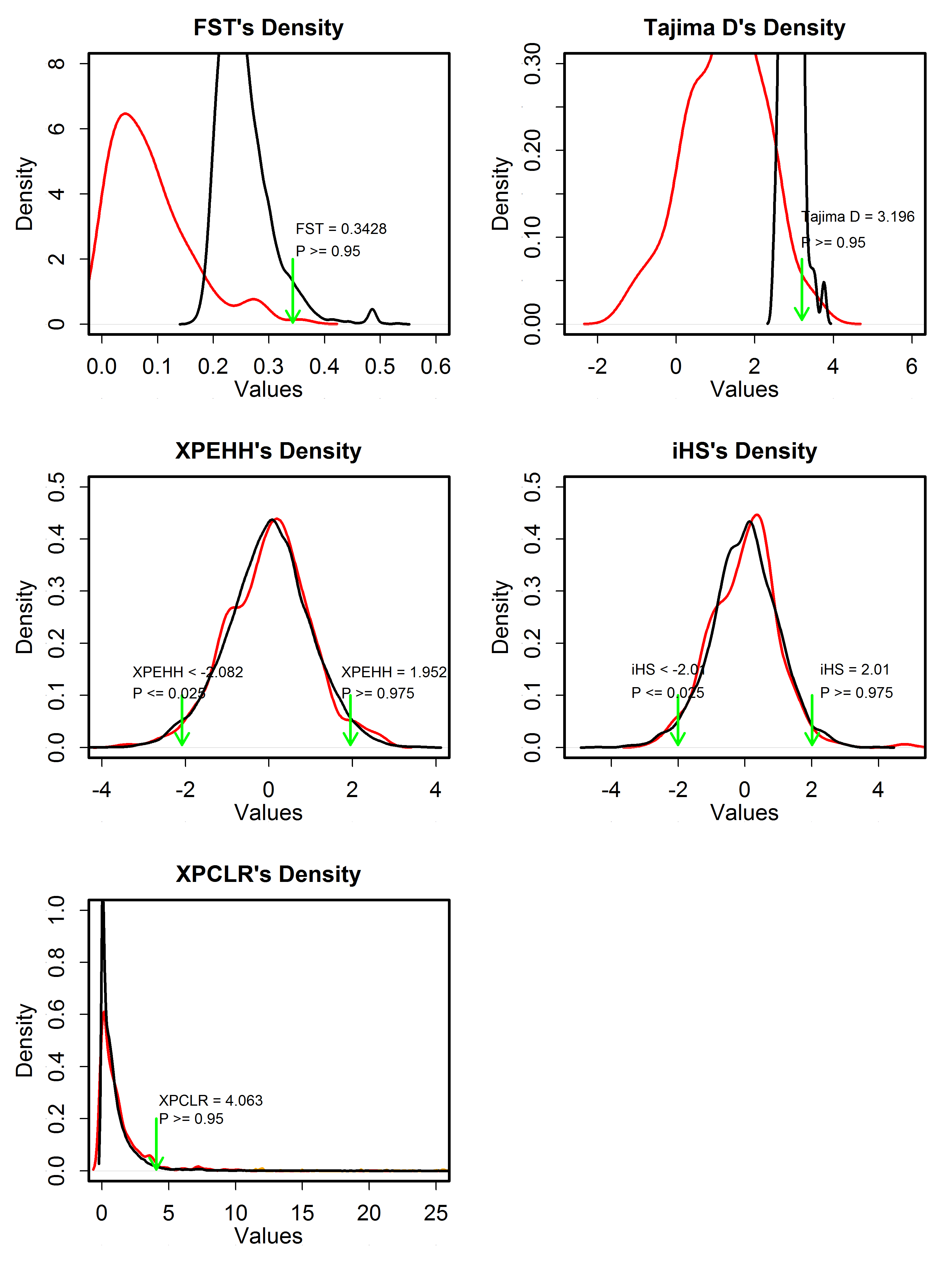

Supplement: Figure S2 — Posterior density of five test statistics. Tajima's D and iHS are for Songliao, XPEHH, XPCLR and Fst are for breed pair of Landrace-Songliao only. (TIFF) [file pone.0094911.s002.tiff]

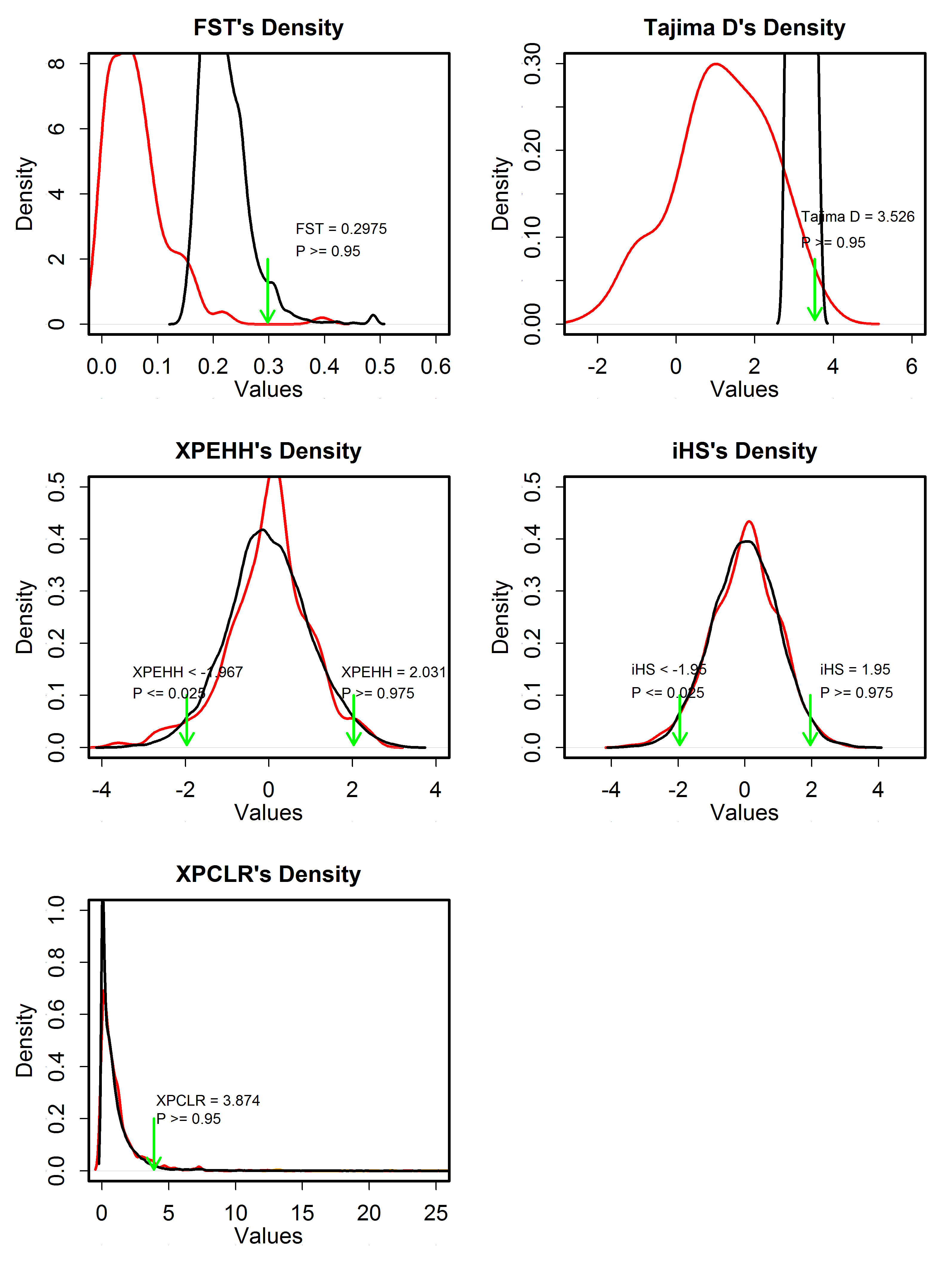

Supplement: Figure S3 — Posterior density of five test statistics. Tajima's D and iHS are for Yorkshire, XPEHH, XPCLR and Fst are for breed pair of Songliao-Yorkshire only. (TIFF) [file pone.0094911.s003.tiff]
