# Supplementary material for: Identification of Selection Footprints on the X Chromosome in Pig
Source: PLoS One. 2014 Apr 16;9(4):e94911. doi: 10.1371/journal.pone.0094911 (PMC3989256; doi:10.1371/journal.pone.0094911)
Supplement: Table S2 — Selection regions identified by more than two methods. (DOCX) [file pone.0094911.s005.docx]

Table S2 selection regions identified by more than two methods.

| Breed | Start | End | Method | Gene name |
| --- | --- | --- | --- | --- |
| Landrace |  |  |  |  |
|  | 23304407 | 24104407 | XPEHH, XPCLR | [MAGEB6](http://www.ensembl.org/Sus_scrofa/Gene/Summary?db=core;g=ENSSSCG00000012187) , [MAGEB5](http://www.ensembl.org/Sus_scrofa/Gene/Summary?db=core;g=ENSSSCG00000012190), [U8](http://www.ensembl.org/Sus_scrofa/Gene/Summary?db=core;g=ENSSSCG00000021185) |
|  | 25176407 | 26178407 | XPEHH, XPCLR | [MAGEB10](http://www.ensembl.org/Sus_scrofa/Gene/Summary?db=core;g=ENSSSCG00000012193) |
|  | 34762255 | 35456407 | XPEHH, XPCLR | [CH242-106K13.1](http://www.ensembl.org/Sus_scrofa/Gene/Summary?db=core;g=ENSSSCG00000012217), [CH242-112D2.1](http://www.ensembl.org/Sus_scrofa/Gene/Summary?db=core;g=ENSSSCG00000012218), [CH242-84C21.3](http://www.ensembl.org/Sus_scrofa/Gene/Summary?db=core;g=ENSSSCG00000030736),  [MAGEB16](http://www.ensembl.org/Sus_scrofa/Gene/Summary?db=core;g=ENSSSCG00000030910), [CXorf22](http://www.ensembl.org/Sus_scrofa/Gene/Summary?db=core;g=ENSSSCG00000012219), CHDC2, U6 |
|  | 85674407 | 85762448 | XPEHH, XPCLR | SNORA18 |
|  | 23304407 | 23762497 | XPCLR,FST | [MAGEB6](http://www.ensembl.org/Sus_scrofa/Gene/Summary?db=core;g=ENSSSCG00000012187), [U8](http://www.ensembl.org/Sus_scrofa/Gene/Summary?db=core;g=ENSSSCG00000030441) |
|  | 25493095 | 26178407 | XPCLR,FST |  |
|  | 22562497 | 22871371 | XPEHH, FST | [CH242-15G6.1](http://www.ensembl.org/Sus_scrofa/Gene/Summary?db=core;g=ENSSSCG00000030881), U6 |
|  | 22962497 | 23762497 | XPEHH, FST | [MAGEB6](http://www.ensembl.org/Sus_scrofa/Gene/Summary?db=core;g=ENSSSCG00000012187), [U8](http://www.ensembl.org/Sus_scrofa/Gene/Summary?db=core;g=ENSSSCG00000021185), [5S_rRNA](http://www.ensembl.org/Sus_scrofa/Gene/Summary?db=core;g=ENSSSCG00000020569) |
|  | 25972376 | 26293095 | XPEHH, FST |  |
| Songliao |  |  |  |  |
|  | 1694407 | 2042528 | XPEHH, XPCLR | [OBP](http://www.ensembl.org/Sus_scrofa/Gene/Summary?db=core;g=ENSSSCG00000012095), [CH242-123G14.1](http://www.ensembl.org/Sus_scrofa/Gene/Summary?db=core;g=ENSSSCG00000012093) |
|  | 2231620 | 2494407 | XPEHH, XPCLR |  |
|  | 121856407 | 1.22E+08 | XPCLR, FST | [OCRL](http://www.ensembl.org/Sus_scrofa/Gene/Summary?db=core;g=ENSSSCG00000012650) |
|  | 2148803 | 2494407 | iHS, XPCLR |  |
|  | 2148803 | 2948803 | XPEHH, iHS | [NLGN4X](http://www.ensembl.org/Sus_scrofa/Gene/Summary?db=core;g=ENSSSCG00000012097),SNORA31 |
|  | 3897515 | 4384968 | XPEHH, iHS | HDHD1, [STS](http://www.ensembl.org/Sus_scrofa/Gene/Summary?db=core;g=ENSSSCG00000026520) |
|  | 92108407 | 92128011 | iHS, Tajima D |  |
|  | 96990740 | 97708406 | iHS, Tajima D | [NXF5](http://www.ensembl.org/Sus_scrofa/Gene/Summary?db=core;g=ENSSSCG00000026976),BEX5, [TCEAL3](http://www.ensembl.org/Sus_scrofa/Gene/Summary?db=core;g=ENSSSCG00000028062),TCEAL2, [ZMAT1](http://www.ensembl.org/Sus_scrofa/Gene/Summary?db=core;g=ENSSSCG00000012512), [ARMCX2](http://www.ensembl.org/Sus_scrofa/Gene/Summary?db=core;g=ENSSSCG00000012510), [ARMCX3](http://www.ensembl.org/Sus_scrofa/Gene/Summary?db=core;g=ENSSSCG00000012509),ARMCX6,CH242-216C10.4,CH242-216C10.1 |
| Yorkshire |  |  |  |  |
|  | 22084407 | 22294683 | XPEHH, XPCLR | CH242-477E15.2, [PCYT1B](http://www.ensembl.org/Sus_scrofa/Gene/Summary?db=core;g=ENSSSCG00000012182),POLA1, [SNORA12](http://www.ensembl.org/Sus_scrofa/Gene/Summary?db=core;g=ENSSSCG00000018651),U6 |
|  | 23902593 | 24248407 | XPEHH, XPCLR |  |
|  | 22084407 | 22871371 | FST,XPCLR | SNORA12, [U6](http://www.ensembl.org/Sus_scrofa/Gene/Summary?db=core;g=ENSSSCG00000020017), [ARX](http://www.ensembl.org/Sus_scrofa/Gene/Summary?db=core;g=ENSSSCG00000020801), [POLA1](http://www.ensembl.org/Sus_scrofa/Gene/Summary?db=core;g=ENSSSCG00000022240), [PCYT1B](http://www.ensembl.org/Sus_scrofa/Gene/Summary?db=core;g=ENSSSCG00000012182), [CH242-15G6.1](http://www.ensembl.org/Sus_scrofa/Gene/Summary?db=core;g=ENSSSCG00000030881), [CH242-202C20.2](http://www.ensembl.org/Sus_scrofa/Gene/Summary?db=core;g=ENSSSCG00000030899),CH242-477E15.2 |
|  | 22962497 | 23762497 | FST,XPCLR | [MAGEB6](http://www.ensembl.org/Sus_scrofa/Gene/Summary?db=core;g=ENSSSCG00000012187), [5S_rRNA](http://www.ensembl.org/Sus_scrofa/Gene/Summary?db=core;g=ENSSSCG00000020569), [U8](http://www.ensembl.org/Sus_scrofa/Gene/Summary?db=core;g=ENSSSCG00000030441) |
|  | 121238570 | 1.22E+08 | FST,XPCLR | [OCRL](http://www.ensembl.org/Sus_scrofa/Gene/Summary?db=core;g=ENSSSCG00000012650), [CH242-218P14.1](http://www.ensembl.org/Sus_scrofa/Gene/Summary?db=core;g=ENSSSCG00000030802), [SMARCA1](http://www.ensembl.org/Sus_scrofa/Gene/Summary?db=core;g=ENSSSCG00000012649), [SNORD112](http://www.ensembl.org/Sus_scrofa/Gene/Summary?db=core;g=ENSSSCG00000020209), [5S_rRNA](http://www.ensembl.org/Sus_scrofa/Gene/Summary?db=core;g=ENSSSCG00000019523) |
|  | 13702407 | 14156031 | iHS, XPCLR | [S100G](http://www.ensembl.org/Sus_scrofa/Gene/Summary?db=core;g=ENSSSCG00000012147), [CTPS2](http://www.ensembl.org/Sus_scrofa/Gene/Summary?db=core;g=ENSSSCG00000012146), [CH242-19L11.2](http://www.ensembl.org/Sus_scrofa/Gene/Summary?db=core;g=ENSSSCG00000030737), [CH242-19L11.1](http://www.ensembl.org/Sus_scrofa/Gene/Summary?db=core;g=ENSSSCG00000012145), [CH242-19L11.3](http://www.ensembl.org/Sus_scrofa/Gene/Summary?db=core;g=ENSSSCG00000030928) |
|  | 7284979 | 8167957 | iHS, XPEHH | [WWC3](http://www.ensembl.org/Sus_scrofa/Gene/Summary?db=core;g=ENSSSCG00000012108), [CLCN4](http://www.ensembl.org/Sus_scrofa/Gene/Summary?db=core;g=ENSSSCG00000024809),XH242-1F19.2, [MID1](http://www.ensembl.org/Sus_scrofa/Gene/Summary?db=core;g=ENSSSCG00000012110),U6 |
|  | 22071371 | 22294683 | XPEHH, FST | [CH242-227G20.3](http://www.ensembl.org/Sus_scrofa/Gene/Summary?db=core;g=ENSSSCG00000031004),CH242-477E15.2,[PCYT1B](http://www.ensembl.org/Sus_scrofa/Gene/Summary?db=core;g=ENSSSCG00000012182), [POLA1](http://www.ensembl.org/Sus_scrofa/Gene/Summary?db=core;g=ENSSSCG00000022240),SNORA12, [U6](http://www.ensembl.org/Sus_scrofa/Gene/Summary?db=core;g=ENSSSCG00000027977) |
|  | 22962497 | 23762497 | XPEHH, FST | [MAGEB6](http://www.ensembl.org/Sus_scrofa/Gene/Summary?db=core;g=ENSSSCG00000012187), [U8](http://www.ensembl.org/Sus_scrofa/Gene/Summary?db=core;g=ENSSSCG00000021185),5S_rRNA |
|  | 25493095 | 26293095 | XPEHH, FST |  |
|  | 4108407 | 4781142 | iHS, Tajima D | [STS](http://www.ensembl.org/Sus_scrofa/Gene/Summary?db=core;g=ENSSSCG00000026520) |
|  | 6018276 | 6508406 | iHS, Tajima D | [CH242-276H23.1](http://www.ensembl.org/Sus_scrofa/Gene/Summary?db=core;g=ENSSSCG00000030749),CH242-236H7.3, [TBL1Y](http://www.ensembl.org/Sus_scrofa/Gene/Summary?db=core;g=ENSSSCG00000012102),U6 |
